# Specific heat, Electrical resistivity and Electronic band structure properties of noncentrosymmetric Th_7_Fe_3_ superconductor

**DOI:** 10.1038/s41598-017-15410-9

**Published:** 2017-11-17

**Authors:** V. H. Tran, M. Sahakyan

**Affiliations:** Institute of Low Temperature and Structure Research, Polish Academy of Sciences, P. O. Box 1410, 50-422 Wrocław, Poland

## Abstract

Noncentrosymmetric superconductor Th_7_Fe_3_ has been investigated by means of specific heat, electrical resisitivity measurements and electronic properties calculations. Sudden drop in the resistivity at 2.05 ± 0.15 K and specific heat jump at 1.98 ± 0.02 K are observed, rendering the superconducting transition. A model of two BCS-type gaps appears to describe the zero-magnetic-field specific heat better than those based on the isotropic BCS theory or anisotropic functions. A positive curvature of the upper critical field *H*_*c*2_(*T*_c_) and nonlinear field dependence of the Sommerfeld coefficient at 0.4 K qualitatively support the two-gap scenario, which predicts *H*_c2_(0) = 13 kOe. The theoretical densities of states and electronic band structures (EBS) around the Fermi energy show a mixture of Th 6d- and Fe 3d-electrons bands, being responsible for the superconductivity. Furthermore, the EBS and Fermi surfaces disclose significantly anisotropic splitting associated with asymmetric spin-orbit coupling (ASOC). The ASOC sets up also multiband structure, which presumably favours a multigap superconductivity. Electron Localization Function reveals the existence of both metallic and covalent bonds, the latter may have different strengths depending on the regions close to the Fe or Th atoms. The superconducting, electronic properties and implications of asymmetric spin-orbit coupling associated with noncentrosymmetric structure are discussed.

## Introduction

Physical properties of noncentrosymmetric superconductors (NSC’s) are particularly interesting as long as the lack of inversion symmetry enhances asymmetric spin - orbit coupling (ASOC), which can remove some degeneracies related to the spin and then the parity conservation becomes violated according the Pauli principle^1–4^. It has been shown that in noncentrosymmetric materials the asymmetric potential gradient ∇*V* is generated by nuclei located at asymmetric crystallographic sites. This potential gradient brings about antisymmetric spin-orbit coupling (ASOC) between electron’s momentum *k* and its spin *σ* presented by the Hamiltonian *H*_*ASO*_ ∝ (∇*V* × *k*).*σ*. Obviously, the ASOC breaks symmetry for an operation of momentum or spin, and splits degenerated bands into two parts of the |*k*↑〉 |−*k*↓〉 and |*k*↓〉 |−*k*↑〉 states, respectively. At the same time, the Fermi surface is partitioned into two subparts with different spin helicities of up or down directions. If the densities of states of these bands differ significantly from one another, the superposition between the states is not possible. In such a situation the pairing state is expected to be a mixture of spin-singlet and spin-triplets states. Moreover, the ASOC also affects the pairing interactions which converse neither spin nor parity. As a result of strongly enhanced pairing interaction the mixed parity pairing could occur too^5^.

Prototype noncentrosymmetric superconductors where the physical properties have experimentally been investigated in more detail are CePt_3_Si^6^ and UIr^7^. In these superconductors, however, the magnetic interaction between the f-electron moments as well as between spins of *f*- and conduction-electrons are very strong, and as a consequence the low-temperature characteristics of the unconventional superconductivity (SC) is conjecturally associated with electron correlation effects. In order to avoid any confusion about the role of non- centrosymmetry constituting the SC phases, it is necessary to investigate noncentrosymmetric superconductors, in which the effects of electron correlation would be negligible. Up to now, most of the investgated noncentrosymmetric supercondutors have conventional s-wave pairing, however, only few like Li_2_Pt_3_B^8,9^, Mo_3_Al_2_C^10,11^, LaNiC_2_^12^ and Re_6_Zr^13^ were reported to show spin-triplet Cooper pairing associated with the missing inversion symmetry.

Recently, we studied low-temperature physical properties of Th_7_Co_3_^14^, which seems to have an anisotropic superconducting gap. Furthermore, electronic band structure calculations within the full-potential linear muffin-tin orbital method of Th_7_Co_3_^14^ and Th_7_Fe_3_^15^ have divulged large splitting energy Δ*E*_*ASOC*_, due to strong ASOC. It is recalled that the superconductivity in series of binaries Th_7_T_3_ (T = Fe, Co, Ir, Rh) has been discovered and investigated many years ago^16–18^. However, to the best of our knowledge, the physical properties of these compounds have not been investigated from the perspective of the influence of the symmetry effect on the superconductivity. In this work, we will continue to study properties of noncentrosymmetric Th_7_Fe_3_ superconductor. The low-temperature behaviour of this compound has been so far explored mainly by specific heat measurements^17,18^. The superconductivity was ascribed to conventional isotropic BCS type with specific heat jump Δ*C*_*p*_/*γT*_*c*_ = 1.43 and upper critical field *H*_*c*2_(0)~6 kOe. However, our measurements of the electrical resistivity and specific heat in this work provide some evidences for two superconducting gaps in Th_7_Fe_3_ Moreover, in this work we also investigate electronic properties using all-electron Full-Potential Linearized Augmented Plane Wave (FP-LAPW) method. We compare the obtained data of Th_7_Fe_3_ with those of very closely-related noncentrosymmetric Th_7_Co_3_ superconductor, aiming to reveal the role of d-electrons in establishing superconductivity in Th_7_T_3_, T = Fe and Co.

## Results and Discussions

### Experimental results

**Figure 1:**
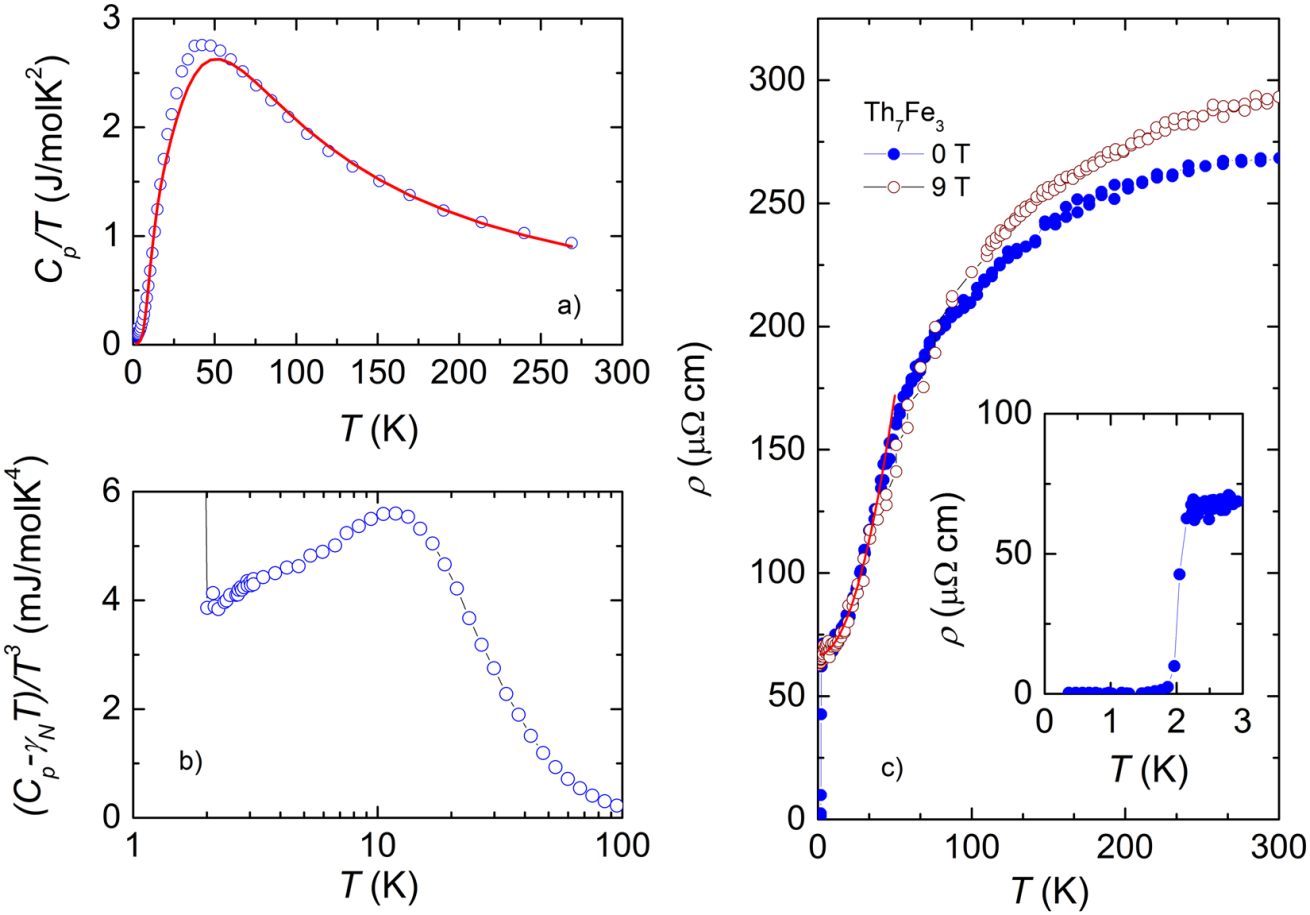
(**a**) Specific heat divided by temperature *C*_*p*_/*T* as a function of temperature. (**b**) The temperature dependence of (*C*_*p*_ − *γ*_*N*_ *T*)/*T* ^3^. (**c**) The temperature dependence of electrical resistivity *ρ*(*T*) at 0 (closed circles) and at 9 T (open circles). The inset shows details near the superconducting transition.

The specific heat *C*_*p*_ measured in the temperature range 2–270 K divided by temperature *T* is shown in Fig. 1(a). The data reach a value of 251.7 J/molK at 270 K, and fairly agree with the Dulong-Petit value of 249.4 J/molK. To analyse the *C*_*p*_(*T*) data we assumed that the total specific heat is a sum of two components: electronic specific heat *C*_*el*_(*T*) = *γT* and lattice specific heat *C*_*ph*_(*T*). The latter consists of Debye *C*_*D*_(*T*) and Einstein *C*_*E*_(*T*) contributions, according to the following equations:

1 $${C}_{D}(T)=9R{n}_{D}{(T/{{\rm{\Theta }}}_{D}^{HT})}^{3}{\int }_{0}^{{{\rm{\Theta }}}_{D}^{HT}/T}\frac{{x}^{4}\,\exp (x)}{{[\exp (x)-\mathrm{1]}}^{2}}dx,$$

2 $${C}_{E}(T)=\frac{3R{n}_{E}\,\exp ({{\rm{\Theta }}}_{E}/T)}{{[\exp ({{\rm{\Theta }}}_{E}/T)-\mathrm{1]}}^{2}},$$

where *R* is the molar gas constant, *n*_*D*_ and *n*_*E*_ are dimensionless Debye-type and Einstein-type vibrators, while ${{\rm{\Theta }}}_{D}^{HT}$ and Θ_*E*_ are the high-temperature Debye and Einstein temperature, respectively. We can justify the presence of optical modes by plotting (*C*_*p*_ − *γ*_*N*_ *T*)/*T* ^3^ *vs*. *T*, depicted in Fig. 1(b). *γ*_*N*_ = 52.7 mJ/molK^2^ is the nornal state Sommerfeld coefficient (see below). One can see a broad maximum at approximately 12 K, which is surely caused by the excess low-frequency vibrations, giving rise to deviation of the specific heat from the Debye model. The best fitting of experimental data with *C*_*el*_(*T*) + *C*_*ph*_(*T*) yields *γ* = 5 mJ/molK^2^, *n*_*D*_ = 8.5, *n*_*E*_ = 1.5, ${{\rm{\Theta }}}_{D}^{HT}$ = 215 and Θ_*E*_ = 55 K. We must concede, however, that the fit (solid line in Fig. 1(a) does not reproduce the temperature dependence of *C*_*p*_/*T* around 35 K correctly. The discrepancy between the experimental and theoretical data exhorts to take into account a more complex phonon density of states than that considered.

In Fig. 1(c) we present the temperature dependence of the electrical resistivity *ρ*(*T*) measured at 0 and 9 T. The zero-field resistivity has a value of *ρ* = 268.3 *μ*Ω cm at room temperature and 62.7 *μ*Ω cm at 2.1 K, resulting in the residual resistivity ratio value of 4.28. We have fitted the resistivity in the temperature range 2–40 K using a simple *ρ*(*T*) = *ρ*_0_ + *AT* ^2^ composed of the residual resistivity *ρ*_0_ and electron-electron scattering contribution *AT* ^2^. The fitting results with *ρ*_0_ = 62.7 *μ*Ω cm and *A* = 0.042 *μ*Ω cm/K^2^ are shown by the solid line. In the normal-state, *ρ*(*T*) of Th_7_Fe_3_, in similar manner to that observed in Th_7_Co_3_^14^, can be characterized by unusual temperature dependence as is compared to those of ordinary metallic alloys. In fact, *ρ*(*T*) has curvature temperature dependence, hence with increasing temperature the resistivity increases in slower manner than that predicted by the Bloch-Grünseisen theory^19^, accounting for acoustic phonons. Above 100 K, *ρ*(*T*) bends downward showing the tendency of saturation. The downward turn in *ρ*(*T*) at high temperatures was found previously in different classes of compounds, e.g., A15-type and Chavrel phase superconductors, 3d and 5d transition metals, high-*T*_*c*_ cuprates^20^, and in some strongly correlated electron systems (SCES)^21^. Unfortunately, there is no generally accepted theory of resistivity saturation for all materials. According to the consideration of Gunnarsson *et al*.^20^, the resistivity of weakly correlated metals may saturate when the inelastic mean-free path tends towards lattice spacing, known as the Ioffe-Regel limit^22^. On the other hand, the saturated resistivity in SCES can be understood based on the Rivier-Zlatic model developed for electron scattering by spin fluctuations at temperatures above spin-fluctuation temperature *T*_*sf*_^23^. For Th_7_Fe_3_ and Th_7_Co_3_ the shape of *ρ*(*T*) curve and a high value of the resistivity at room temperature would be consistent with feature due to a strong coupling of conduction electrons to fluctuating *d*-electron spins.

The resistivity in the temperature range 0.4–3.0 K is displayed in the inset of Fig. 1(c). Evidently, *ρ*(*T*) discloses a sharp drop at 2.1 K and vanishes at *T*_*c*_ = 1.95 K, revealing the transition into a superconducting state. Using a 50 % normal-state resistivity criterion, the critical temperature is estimated as 2.05 K. The transition width Δ*T*_*c*_ defined as the difference of *T* at 10% to 90% of resistivity at the transition is 0.15 K. We note that our experimental *T*_*c*_ is the same as that in ref.^17^ but is a little higher than that previously reported *T*_*c*_ = 1.86 K^16^.

**Figure 2:**
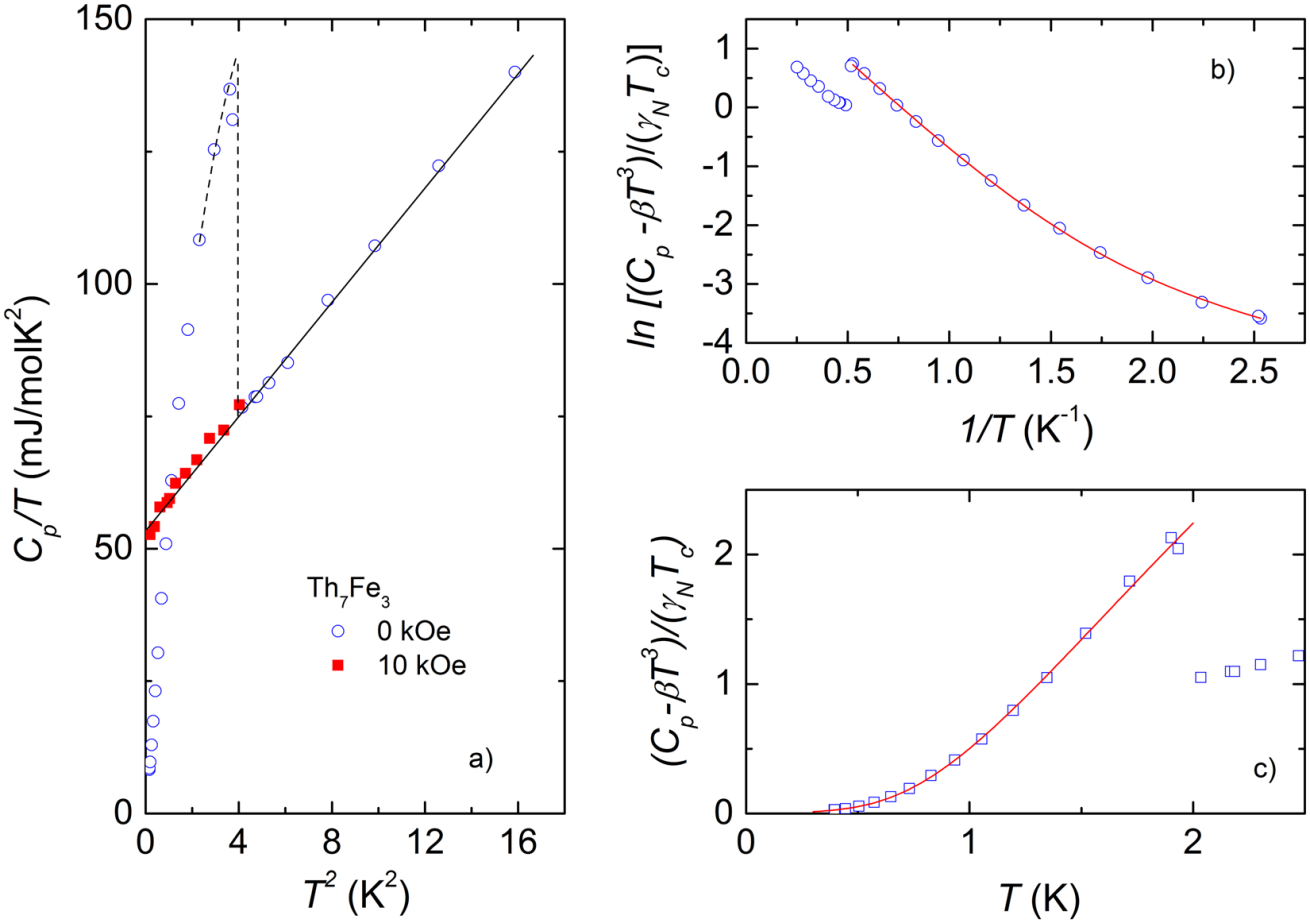
(**a**) Low-temperature *C*_*p*_/*T* at zero (open circles) and 10 kOe field (closed squares) as a function of *T* ^2^. (**b**) The normalized electronic specific heat of Th_7_Fe_3_ (*C*_*p*_ − *βT* ^3^)/(*γ*_*N*_ *T*_*c*_) in a log scale *vs*. the inverse temperature, 1/*T*. The solid line is fit of two-gap model to the data. (**c**) The experimental and theoretical data (*C*_*p*_ − *βT* ^3^)/(*γ*_*N*_ *T*_*c*_) *vs*. *T* of Th_7_Fe_3_.

A strong proof for the bulk superconductivity in Th_7_Fe_3_ is the specific heat jump at zero field shown in Fig. 2(a). The critical temperature is taken as the position of the half height of the *C*_*p*_/*T*-jump, *T*_*c*_ = 1.98 ± 0.02 K. We calculated the specific heat jump Δ*C*_*p*_(*T* = *T*_*c*_) as the difference between the *C*_*p*_ at *T*_*c*_ and the normal state specific heat (illustrated by the dashed line in Fig. 2(a)). The normalized jump Δ*C*_*p*_/(*γ*_*N*_ *T*_*c*_) amounts to 1.21, being substantially larger than 1.01 in Th_7_Co_3_^14^. We notice that the observed specific heat jump in both these compounds is much reduced as compared to the BCS value of 1.43^24^.

To estimate the electronic contribution to the specific heat we considered 10 kOe-*C*_*p*_/*T* (closed squares) for *T* < *T*_*c*_ and zero-field *C*_*p*_/*T* (open circles) for *T* > *T*_*c*_. The least-squares fitting of experimental *C*_*p*_-data with a sum of an electronic *γ*_*N*_ *T* and lattice *βT* ^3^ contributions yields the Sommerfeld coefficient *γ*_*N*_ = 52.7(1) mJ/molK^2^ and the Debye constant *β* = 5.51 mJ/molK^4^. The best fitting result is shown by the solid line in Fig. 2(a). Using the experimental Sommerfeld coefficient *γ*_*N*_ the band structure Density of states at Fermi level *N*(*E*_*F*_) can be deduced from the formula:

3 $$N({E}_{F})=\frac{3{\gamma }_{N}}{{\pi }^{2}{k}_{B}^{2}{N}_{A}}\mathrm{.}$$

*k*_*B*_ is Boltzmann’s constant, *N*_*A*_ is Avogadro’s number and *N*(*E*_*F*_) is found to be 22.36 st/eV.f.u. From the *β*-value, and taking into consideration the relation:

4 $${{\rm{\Theta }}}_{D}^{LT}={(n\frac{12{\pi }^{4}R}{5\beta })}^{\mathrm{1/3}},$$

where *n* = 10 is the number of atoms per mole, we calculated low-temperature Debye temperature ${{\rm{\Theta }}}_{D}^{LT}$ = 152.2 K. Having the critical temperature *T*_*c*_ and Debye temperature ${{\rm{\Theta }}}_{D}^{LT}$, we can evaluate the electron-phonon coupling constant ${\bar{\lambda }}_{{\rm{el}}-{\rm{ph}}}$ using the McMillan’s equation^25^:

5 $${\bar{\lambda }}_{{\rm{el}}-{\rm{ph}}}=\frac{1.04+{\mu }^{\ast }\,\mathrm{ln}\,\frac{{{\rm{\Theta }}}_{D}^{LT}}{1.45{T}_{c}}}{(1-0.62{\mu }^{\ast })\,\mathrm{ln}\,(\frac{{{\rm{\Theta }}}_{D}^{LT}}{1.45{T}_{c}})-1.04},$$

where μ* is repulsive Coulomb parameter, which is typically given in the range 0.1–0.15. Assuming μ* = 0.125 we obtained ${\bar{\lambda }}_{{\rm{el}}-{\rm{ph}}}$ = 0.59, which is practically not different from 0.56 for Th_7_Co_3_^14^.

According to the BCS description of the electronic specific heat, the superconducting energy gap Δ_0_ is given by an equation^26^:

6 $${C}_{{\rm{el}}}(T)=A{\gamma }_{N}{T}_{c}\,\exp \,(-\frac{{{\rm{\Delta }}}_{0}}{{k}_{B}T}),$$

where *A* is a constant. In order to check the prediction of the BCS theory for the superconductivity one should plot the normalized electronic specific heat (*C*_*p*_ − *βT* ^3^)/(*γ*_*N*_ *T*_*c*_) in a log scale *vs*. the inverse of temperature, 1/*T*. For Th_7_Fe_3_ such a plot is shown in Fig. 2(b). Apparently, a straight line cannot be used to describe the data between 0.4–*T*_*c*_ and this observation allows us to propose that the superconductivity in Th_7_Fe_3_ is not a classic isotropic s-wave BCS-type. Adapting the same treatment of data as was previously utilized for closely related Th_7_Co_3_ compound, we fitted the specific heat data using two models of non-isotropic gap structure: a) two-gap and b) anisotropic gap, respectively. In the two-gap model, electronic specific heat is assumed to be the sum of two contributions with different values of gaps (Δ_1_, Δ_2_) and electronic specific heat coefficients (*γ*_1_, *γ*_2_). The electronic specific heat data of the Th_7_Fe_3_ superconductor was fitted with the equation:

7 $${C}_{{\rm{el}}}(T)=A{\gamma }_{N}{T}_{c}\,[x\,\exp \,(-\frac{{{\rm{\Delta }}}_{1}}{{k}_{B}T})+\mathrm{(1}-x)\,\exp \,(-\frac{{{\rm{\Delta }}}_{2}}{{k}_{B}T})]\mathrm{.}$$

In fittings, *γ*_*N*_ = 52.7 mJ/molK^2^ and *T*_*c*_ = 1.98 K were kept constant, and we obtained the best fit with Eq. 7 for the following parameters *A* = 10.82, Δ_1_/*k*_*B*_ = 3.22 K, Δ_2_/*k*_*B*_ = 0.75 K and *x* = 0.985. The result of fit is illustrated by the solid line in Fig. 2(b and c). Within anisotropic gap scenario, we examined the superconducting state electronic specific heat, employing the same equations as were used for Th_7_Co_3_^14^. However, the fittings of experimental data of Th_7_Fe_3_ to anisotropic gap model did not give satisfactory result.

We calculated the thermodynamic critical field *H*_*c*_(*T*) according to equations:

8 $$-\frac{1}{2}{\mu }_{0}V{H}_{c}^{2}(T)={\rm{\Delta }}F(T)={\rm{\Delta }}U(T)-T{\rm{\Delta }}S(T),$$

where *μ*_0_ is the magnetic constant, *V* is the unit cell volume. The variation of internal energy Δ*U*(*T*) can be obtained by integrating the difference of the specific heat in the superconducting *C*_*s*_(*T*) and in the normal *C*_*n*_(*T*) states:

9 $${\rm{\Delta }}U(T)={\int }_{T\mathrm{=0}}^{{T}_{c}}[{C}_{s}(T^{\prime} )-{C}_{n}(T^{\prime} )]dT^{\prime} ,$$

while the variation of entropy Δ*S*(*T*) is obtained via the difference of the entropies in the normal and in the superconducting states:

10 $${\rm{\Delta }}S(T)={\int }_{T\mathrm{=0}}^{{T}_{c}}\frac{{C}_{s}(T^{\prime} )-{C}_{n}(T^{\prime} )}{T^{\prime} }dT^{\prime} \mathrm{.}$$

**Figure 3:**
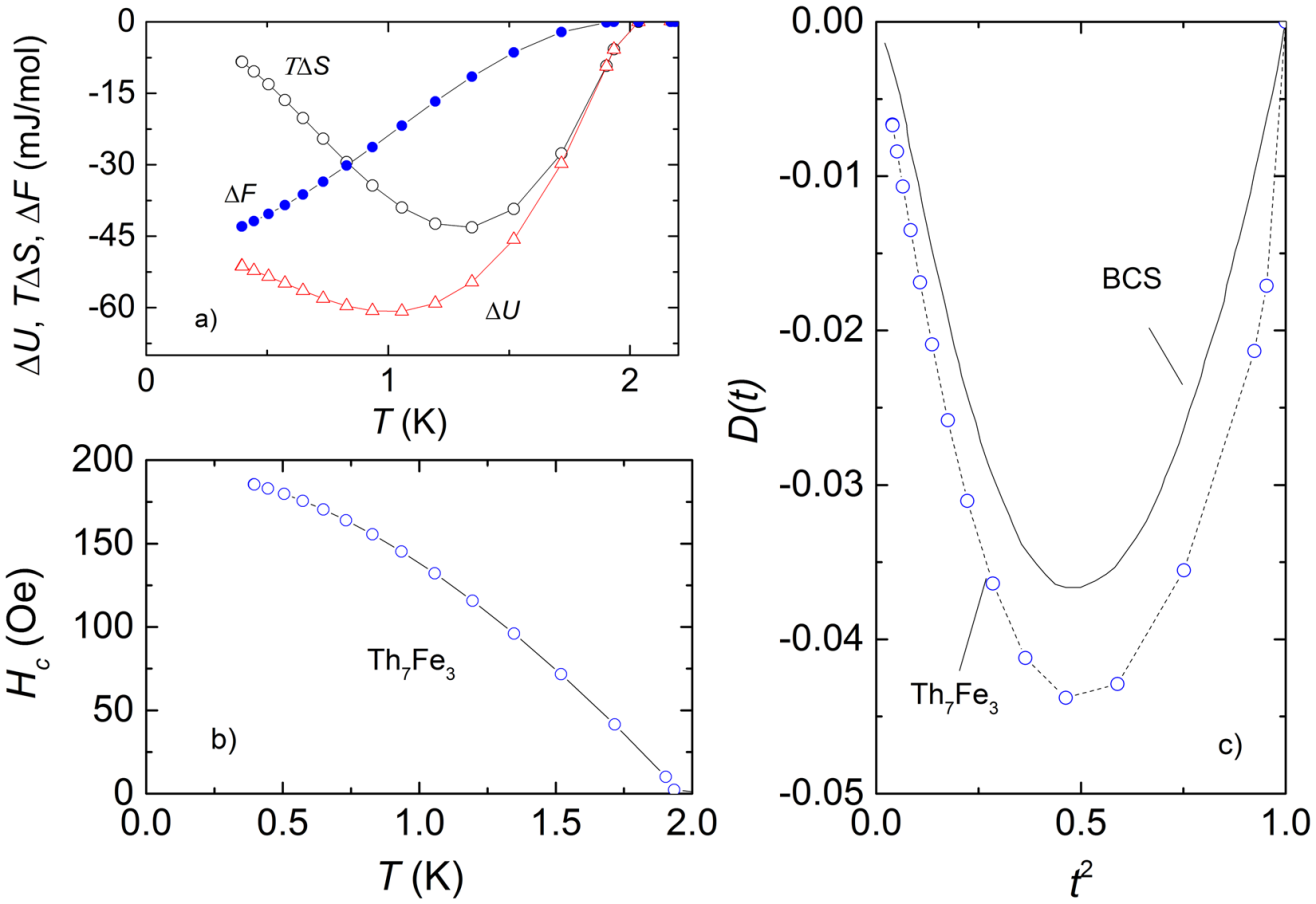
(**a**) Internal energy difference Δ*U*(*T*), entropy difference multiplied by the temperature *T*Δ*S*(*T*) and free energy Δ*F*(*T*). (**b**) Thermodynamic critical field obtained by integration of specific heat data using Eqs [8–10]. (**c**) The deviation function *D*(*t*) = *H*_*c*_(*t*)/*H*_*c*_(0) − (1 − *t* ^2^) vs. *t* ^2^, where *t* = *T*/*T*_*c*_.

The calculated temperature dependencies of the internal energy Δ*U*(*T*), entropy multiplied by the temperature *T*Δ*S*(*T*) and free energy Δ*F*(*T*) are shown in Fig. 3(a) while *H*_*c*_(*T*) is shown in Fig. 3(b). In order to evaluate the value of *H*_*c*_(0) at 0 K we used Taylor expansion of thermodynamic critical field *H*_*c*_(*T*)^27^:

11 $${H}_{c}(T)={H}_{c}\mathrm{(0)[1}-b{(T/{T}_{c})}^{2}+\mathrm{(1}-b)(T/{T}_{c}{)}^{4}],$$

where *b* is a fitting parameter. For (*T*/*T*_*c*_)^2^ up to 0.6 we observed linear dependence of *H*_*c*_(*T*)/[1 − (*T*/*T*_*c*_)^2^] *vs* (*T*/*T*_*c*_)^2^ with the intercept *H*_*c*_(0) = 194.6 Oe and the slope of −34.5, giving *b* = 1.177. Taking the electronic specific heat coefficient *γ*_*V*_ = 3361.43 erg/cm^3^ K^2^ and putting the fitted value of the thermodynamic critical field to the equation:

12 $${H}_{c0}^{2}\mathrm{(0)}=(\frac{3{\gamma }_{V}}{2{\pi }^{2}{k}_{B}^{2}})\,{{\rm{\Delta }}}_{0}^{2},$$

yield superconducting gap Δ_0_/*k*_*B*_ = 2.43 K, which is much smaller than Δ_1_/*k*_*B*_ = 3.22 K found above. Another noticeable feature of the superconductivity in Th_7_Fe_3_ is presented by the behaviour of deviation function *D*(*t*) = *H*_*c*_(*t*)/*H*_*c*_(0) − (1 − *t* ^2^), with *t* = *T*/*Tc*. It is seen from Fig. 3(c) the deviation function lies below the BCS curve, thus electron-phonon coupling in the studied compound is weak.

**Figure 4:**
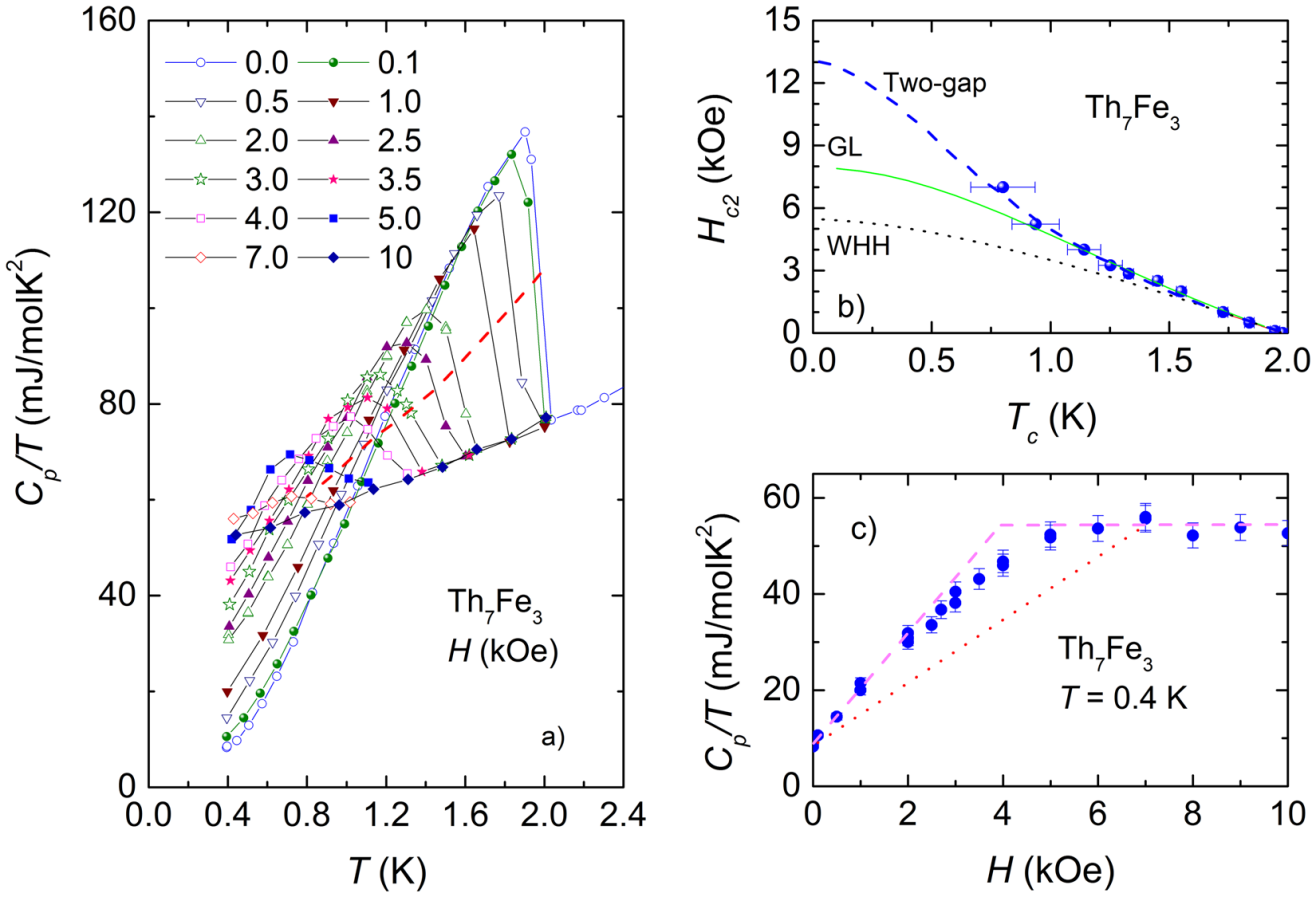
(**a**) Temperature dependence of *C*_*p*_/*T* of Th_7_Fe_3_ in several magnetic fields up to 10 kOe. The dashed line is a guide for the eye, showing the suppression of *T*_*c*_ by the applied magnetic field. (**b**) Temperature dependence of the upper critical field in Th_7_Fe_3_. The dotted, solid and dashed lines are theoretical data based on the WHH, GL and two-gap models, respectively. Note that the WHH curve is not sensitive to the choice of *λ*_*so*_ > 10. (**c**) Field dependence of the ratio *C*_*p*_/*T* at 0.4 K. The dotted and dashed lines are guides to the eye.

Low-temperature specific heat data for Th_7_Fe_3_ in several magnetic fields up to 10 kOe are plotted as *C*_*p*_/*T vs*. *T* ^2^ in Fig. 4(a). An increasing applied field causes broadening of superconducting transition and lowers ${{C}_{p}/T|}_{{T}_{c}}$-jump. One recognizes that the suppression of superconductivity accompanies steady increase of *C*_*p*_/*T* ratio at 0.4 K. We determined the upper critical field *H*_*c*2_ dependence on *T*_*c*_ as illustrated by dashed line. The obtained *H*_*c*2_(*T*_*c*_) and ${{C}_{p}/T(H)|}_{0.4K}$ data are presented in Fig. 4(b and c), respectively. The slope *dH*_*c*2_/*dT* near *T*_*c*_ was found to be approximately −3.96 kOe/K. Using the Werthamer-Helfand-Hohenberg (WHH) formula for a type-II dirty superconductor, ${H}_{c2}\mathrm{(0)}={-0.69{T}_{c}(d{H}_{c2}/dT)|}_{{T}_{c}}$ ^28^, we estimated the zero temperature upper critical field *H*_*c*2_(0) = 5.4 kOe. The *H*_*c*2_(*T*_*c*_) curve in the whole temperature range 0 − *T*_*c*_ can be simulated using the digamma function^28^:

13 $$\begin{array}{rcl}\mathrm{ln}\,\frac{1}{t} & = & (\frac{1}{2}+\frac{i{\lambda }_{so}}{4\gamma })\,\psi \,(\frac{1}{2}+\frac{\bar{h}+{\lambda }_{so}\mathrm{/2}+i\gamma }{2t})\\  &  & +\,(\frac{1}{2}-\frac{i{\lambda }_{so}}{4\gamma })\,\psi \,(\frac{1}{2}+\frac{\bar{h}+{\lambda }_{so}\mathrm{/2}-i\gamma }{2t})-\psi (\frac{1}{2}),\end{array}$$

where *t* = *T*/*T*_*c*_, $\gamma ={[(\alpha \bar{h}{)}^{2}-{({\lambda }_{so}\mathrm{/2)}}^{2}]}^{\mathrm{1/2}}$, $\bar{h}=\frac{4{H}_{c2}}{{{\pi }^{2}(-d{H}_{c2}/dt)|}_{t=1}}$ and $\alpha ={-\mathrm{0.528(}d{H}_{c2}/dT)|}_{Tc}$ is the Maki parameter. For *α* = 0.21 and *λ*_*so*_ = 10 we obtained the dotted line, which presents the best description of the WHH model to the experimental data. Unfortunately, as can be seen in the figure, the WHH model has failed to describe the *H*_*c*2_(*T*_*c*_) dependence of Th_7_Fe_3_. In fact, the theoretical WHH values are very significantly underestimated as compared with the experimental ones.

A greater value of the 0 K upper critical field *H*_*c*2_(0) can be obtained with the help of the Maki theory^29^:

14 $${H}_{c2}\mathrm{(0)}=\alpha {H}_{p}\mathrm{(0)/}\sqrt{2},$$

where *H*_*P*_(0) is the zero temperature Pauli limiting field. The latter quantity is closely associated with the BCS value for paramagnetic limiting field via the relation ${H}_{p}\mathrm{(0)}={H}_{p}^{BCS}\sqrt{1+{\lambda }_{{\rm{el}}-{\rm{ph}}}}$, where ${H}_{p}^{BCS}$ = 1.83 *T*_*c*_^30^. Taking *α* = 0.21, *λ*_el−ph_ = 0.59 and *T*_*c*_ = 1.98 K, we get *H*_*c*2_(0) = 6.8 kOe. This value is in close agreement with the value of *H*_*c*2_(0) = 8 kOe derived from the Ginzburg-Landau (GL) formula:

15 $${H}_{c2}(t)={H}_{c2}\mathrm{(0)}\frac{1-{t}^{2}}{1+{t}^{2}}\mathrm{.}$$

The above equation is simply deduced from well known relations:

16 $$\begin{array}{rcl}{H}_{c2}\mathrm{(0)} & = & \frac{{{\rm{\Phi }}}_{0}}{2\pi {\xi }_{GL}{\mathrm{(0)}}^{2}},\,{\rm{and}}\\ \,{\xi }_{GL}(t) & = & \sqrt{\mathrm{(1}+{t}^{2}\mathrm{)/(1}-{t}^{2})},\end{array}$$

where Φ_0_ is the magnetic flux quantum and *ξ*_*GL*_ is the Ginzburg-Landau coherence length. The fit of Eq. 15 to experimental data is shown by the solid line in Fig. 4(b). However, the GL model is insufficient to reproduce the convex curvature of the experimental *H*_*c*2_(*T*_*c*_) data below 1.2 K. There are several possible reasons for an enhancement and concave-upward behaviour of *H*_*c*2_(*T*_*c*_)^31^, including twisting of electron orbits by a magnetic field^32^, dimensional crossover^33^ and multi-gap structure^34^. The first mechanism was considered by Lebed^32^ for low-dimensional organic superconductors, in which the twisting of electron orbits by a magnetic field was assumed to be important. It was noted that the upward curvature in *H*_*c*2_(*T*_*c*_) is expected to occur below a characteristic temperature T* < *T*_*c*_ and only for the plane of applied magnetic field. Our measurements were conducted on polycrystalline samples and the studied compound is a 3D material, therefore the low-dimensional effect has nothing to do with the observed anomaly of *H*_*c*2_(*T*_*c*_). The mechanism based on multiple-gap structure has been used to interpret *H*_*c*2_(*T*_*c*_) for numerous two-gap superconductors, e.g., MgB_2_^35^, YNi_2_B_2_C, LuNi_2_B_2_C^36^, LaNiC_2_^37^ and FeAs-based^31,38^. Assuming that the superconductivity in Th_7_Fe_3_ is interwoven with two-band nature, we are able to simulate *H*_*c*2_(*T*_*c*_) dependence (dashed line in Fig. 4(b)) using the formula developed by Gurevich^34^:

17 $${a}_{0}[\,\mathrm{ln}\,t+U(h)]\,[\,\mathrm{ln}\,t+U(\eta h)]+{a}_{2}[\,\mathrm{ln}\,t+U(\eta h)]+{a}_{1}[\,\mathrm{ln}\,t+U(h)]=\mathrm{0,}$$

where *a*_0_, *a*_1_ and *a*_2_ are parameters associated with intraband *λ*_11_, *λ*_22_ and interband *λ*_12_, *λ*_21_ couplings, *η* = *D*_2_/*D*_1_ is the ratio of diffusivities of bands and *U*(*x*) = *ψ*(1/2 + *x*) − *ψ*(1/2) is the difference of di-gamma functions. We must admit that though the agreement between the experimental and theoretical data seems to be satisfactory, there remains questionable reliability of obtained fitting parameters since the fit was done for large number of fitting parameters. Nonetheless, the extrapolated zero-temperature upper critical field *H*_*c*2_(0) = 13 kOe, corresponding to *ξ*_*GL*_ = 15.9 nm seems to be reasonable since *ξ*_*GL*_ has the same order of magnitude as that found via evaluation of the equation^39^:

18 $$\xi \mathrm{(0)}=\frac{8.57\times {10}^{-7}}{\sqrt{{\gamma }_{erg}{\rho }_{n}{T}_{c}}}\mathrm{.}$$

Using *γ*_*V*_ = 3361.43 erg/cm^2^ K^2^ and the normal state resistivity *ρ*_*n*_ = 62.7 × 10^−6^ Ω cm, we obtained *ξ*(0) = 13.2 nm.

Yet, we can evaluate the Ginzburg-Landau penetration depth from the values of the upper and thermodynamic critical fields:

19 $${\lambda }_{GL}=\sqrt{\frac{{{\rm{\Phi }}}_{0}{H}_{c2}\mathrm{(0)}}{4\pi {H}_{c}{\mathrm{(0)}}^{2}}}\mathrm{.}$$

Putting *H*_*c*2_ = 13 kOe and *H*_*c*_(0) = 194.6 Oe into eq. 19 we get *λ*_*GL*_ = 751.4 nm and the Ginzbug-Landau parameter *κ* takes value of *κ* = *λ*_*GL*_/*ξ*_*GL*_~47. Alternatively, penetration depth is determined using the formula^39^:

20 $$\lambda =6.42\times {10}^{-3}\sqrt{\rho /{T}_{c}}\mathrm{.}$$

Using eq. 20, we get *λ* = 361.2 nm, which together with *ξ*(0) = 13.2 nm yields *κ* = 27.3. The estimated *κ* values establish Th_7_Fe_3_ to be a superconductor of type-II.

### Theoretical results

**Figure 5:**
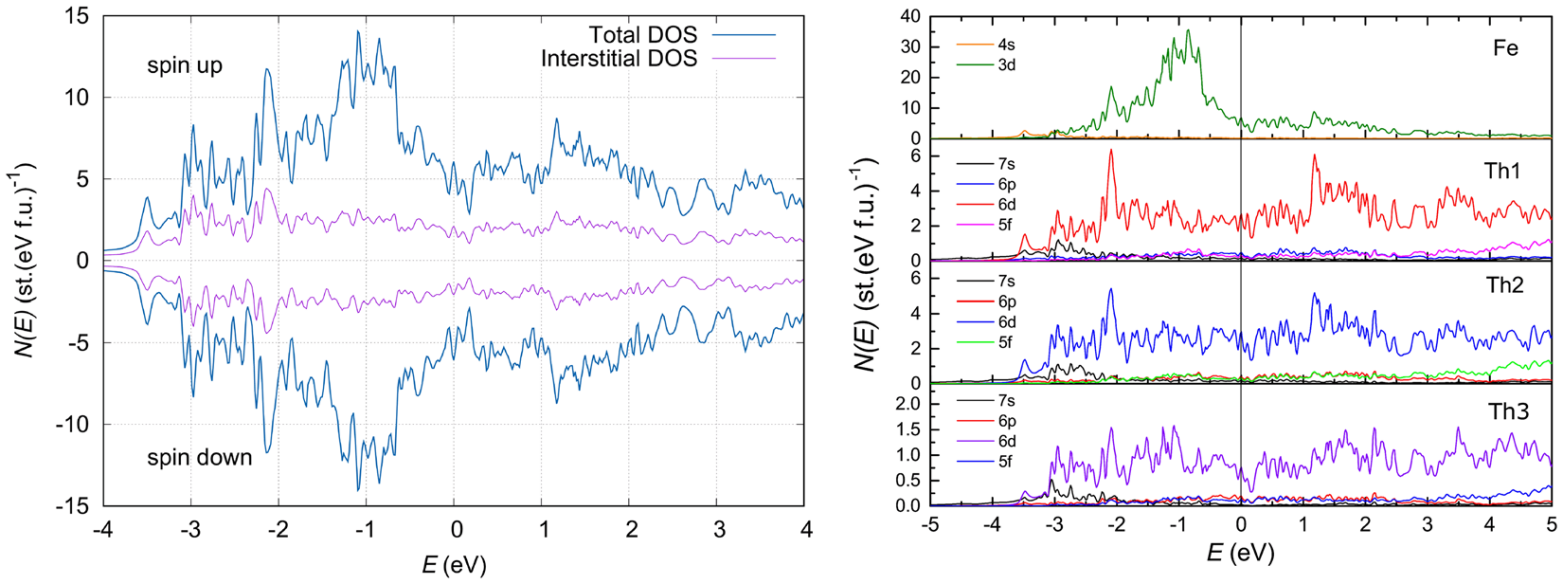
Left-side panel: The total (thick line) and interstitial (thin line) DOS of Th_7_Fe_3_. Right-side panel: The partial DOS contributions from the Fe and Th atoms per one spin direction.

In the left-side panel of Fig. 5 we depict the total and interstitial DOS of Th_7_Fe_3_. The data were obtained for spin polarization within the fully relativistic (FR) approximation. In the figure, we observe no spin polarization effect, thus implying a non-magnetic the ground state of Th_7_Fe_3_, even in the presence of spin-orbit interaction. The finding is in agreement with experimental data collected down to 0.4 K. Resemblance of the total DOS with those from FP-LMTO calculations^15^ is high, in respect of both the DOS values at the Fermi energy *E*_*F*_ and DOS feature below *E*_*F*_. Here, *N*(*E*_*F*_) amounts approximately to 20 st./(eV. f.u) and there exists peak structure at around −1 eV. Its akin to the Van Hove singularity often observed in superconductors. The relative contributions from the muffin-tin sphere and interstitial region to the total DOS can be evaluated by comparing the calculated values of the total and interstitial DOS’s. If we focus on the data around *E*_*F*_ we see an sizeable contribution from the interstitial region, so the overlap of orbitals is expected to be essential. Obviously, the main contribution to the total DOS below 0.5 eV comes from the muffin-tin spheres, where the orbitals around the atoms are atomic-like. In right-side panel of Fig. 5 we show the partial DOS calculated for one spin direction. We perceive that the contributions of 3*d* and 6*d*-electrons orbitals at the *E*_*F*_ are almost equal and they dominate the DOS. The DOS derived from the remaining orbitals are negligible, thus we would expect important performance of the mixture of 3d and 6d-orbitals for the superconductivity of Th_7_Fe_3_.

**Figure 6:**
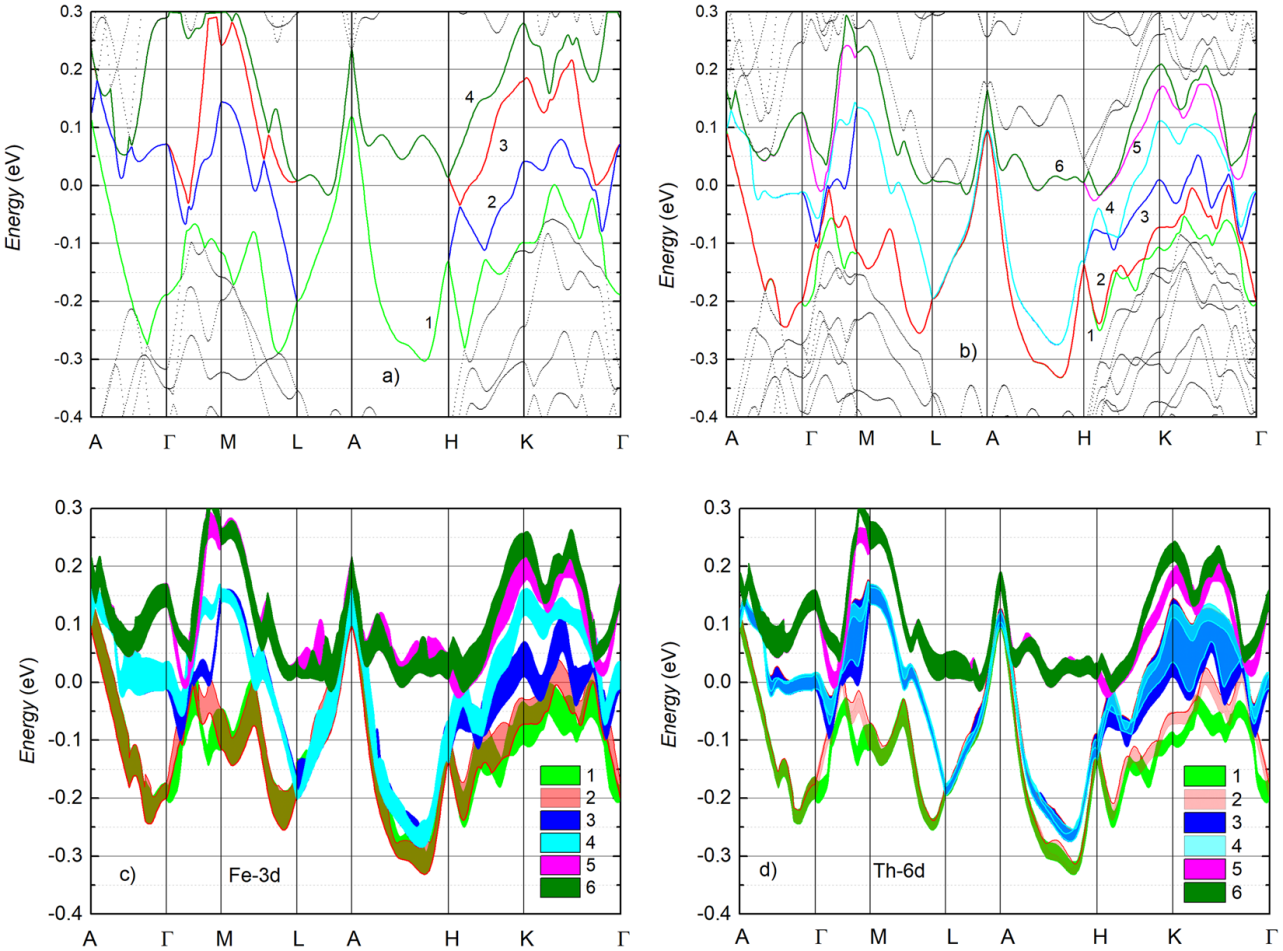
Electronic band structures calculated without (**a**) and with spin-orbit coupling (**b**) effect. The bands crossing *E*_*F*_ are colored only. The orbital-projected band structures of the Fe-3d and Th-6d electrons from FR calculations are shown in (**c** and **d**), respectively. The width of lines represent the projected weight to angular momentum of the orbitals. *A* (0.0, 0.0, 0.5), Γ (0.0, 0.0, 0.0), *M* (0.5, 0.0, 0.0), *L* (0.5, 0.0, 0.5), *H* (2/3, 1/3, 0.5) and *K* (2/3, 1/3, 0.0) are points of high symmetry in the first Brillouin zone of hexagonal lattice.

In Fig. 6(a and b) we compare electronic band structures (EBS) obtained without and with spin-orbit coupling. Evidently, the calculation without spin-orbit coupling conveys a quite lucent electronic structure with several bands crossing *E*_*F*_. This feature together with fairly flat and closely lying bands in the energy range 1.5–0.5 eV below *E*_*F*_ (not shown here) reflect the behaviour of *N*(*E*) curve (see Fig. 5a). Looking at Fig. 6(a) we can see that the band 1 (green color) crosses *E*_*F*_ in the directions *A* − Γ, *A* − *L* and *A* − *H*. The band 2 (blue color), 3 (red color) and 4 (olive) have hole structure at both *A* and Γ points. The dominance of hole bands is seen in the compound without spin-orbit coupling. When the spin-orbit coupling was included in the calculations the electronic band structure gets more complex and as much as six bands with the bandwidth of 0.303–0.385 eV crossing *E*_*F*_ can be recognized. We remark that the splitting into spin polarized bands is highly anisotropic in momentum space, namely, the band structure along the directions Γ − *M*, *H* − *K* and *K* − Γ exhibits a very strong dispersion, in comparison with that along the *A* − Γ completely lacking split. This behaviour may account for a combined outcome of relativistic effect and ASOC. It is worthwhile to highlight that the spin-orbit coupling rearranges levels of the band energy. The energy of bands 1–2 and 3–4 (Fig. 6b) as respectively compared to those of bands 1 and 2 (Fig. 6a) becomes lowered. On the other hand, the bands 5 and 6 (Fig. 6b) are weakly changed versus band 3 (Fig. 6a). In contrast, the band 4 (Fig. 6a) is pushed upwards and no longer crosses the *E*_*F*_ (Fig. 6b).

To gain insights into the contributions of 3d- and 6d-electrons, the orbital-projected band structures of the Fe and Th atoms are shown in Fig. 6(c and d), respectively. At first glance, the overall features of the 3d- and 6d-electron band structures are similar. This observation indicates a robust mixture of 3d and 6d orbitals in the energy range around *E*_*F*_. There are differences between projected weights, which are distinctly bigger for those of the 3d orbitals and may suggest that the 3d-electrons are more localized. An inspection of Fig. 6(c and d) reveals that three kinds of electronic bands exist nearby the Fermi level. There are two bands, denoted as 1 and 2, hole-like at the *A* point, but electron-like around the Γ point. These bands elucidate the metallic nature of the compound. Other two bands crossing the *E*_*F*_, denoted as 5 and 6, have hole-like properties around both the *A* and Γ points. The remaining two bands, denoted as 3 and 4, have both hole- and electron-like character at Γ. Clearly, the ASOC induces two types of carries through lowering energy levels of partial bands as compared with dominant holes in the case without SOC. We believe that the multiband structure induced by ASOC associated with lack of inversion symmetry possibly entails multiple-gap superconductivity in the studied material.

**Figure 7:**
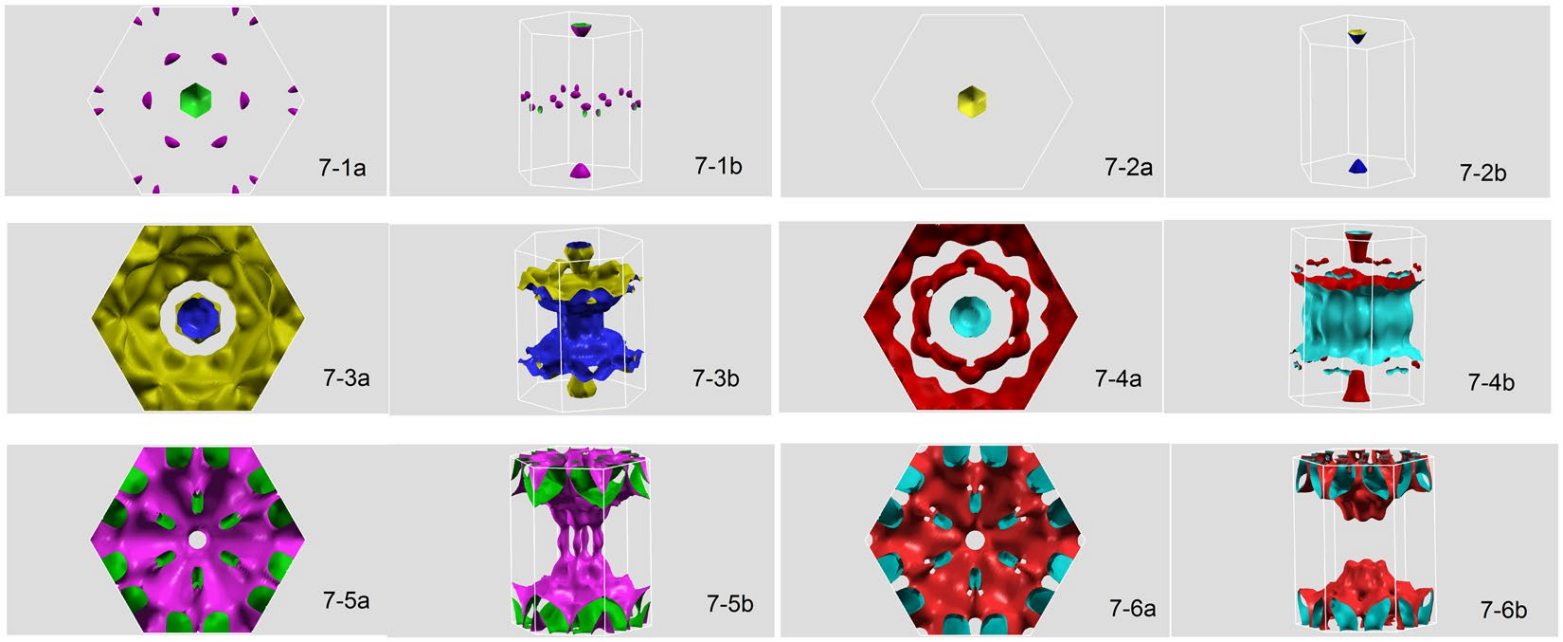
Fermi surfaces of bands crossing *E*_*F*_.

Fermi surfaces (FS) in the first Brillouin zone of the six bands crossing the Fermi energy are presented in Fig. 7. The notation a and b corresponds to FS view from top and in 3D forms, respectively. We would like to emphasise that the FS’s shown in Fig. 7(1), (3) and (5) are similar to those from SR calculations (not shown here), though there are some noticeable differences due to spin-orbit coupling. For example, for FS in Fig. 7(1) the pocket at *K* point becomes split onto two pockets around this point. Thereafter, for FS in Fig. 7(3), the holelike band around Γ point in the SR approach turns into electron-like in the FR calculation. Finally, for FS in Fig. 7(5), we see that the six tubes along the *A* − Γ direction alter to more and more slender shapes. Obviously, the FS’s shown in Fig. 7(2), (4) and (6) do not appear in the SR calculations, thus split FS properties must to be associated with SOC. It should be kept in mind that crystal symmetry plays a role in the formation of FS’s, in particular, it may impinge on the properties of individual FS sheets and anisotropy of FS’s. For Th_7_Fe_3_, we discern that FS’s viewed from top are seen to be essentially symmetric, while FS’s in every planes embodying the *A* − Γ line are highly anisotropic.

**Figure 8:**
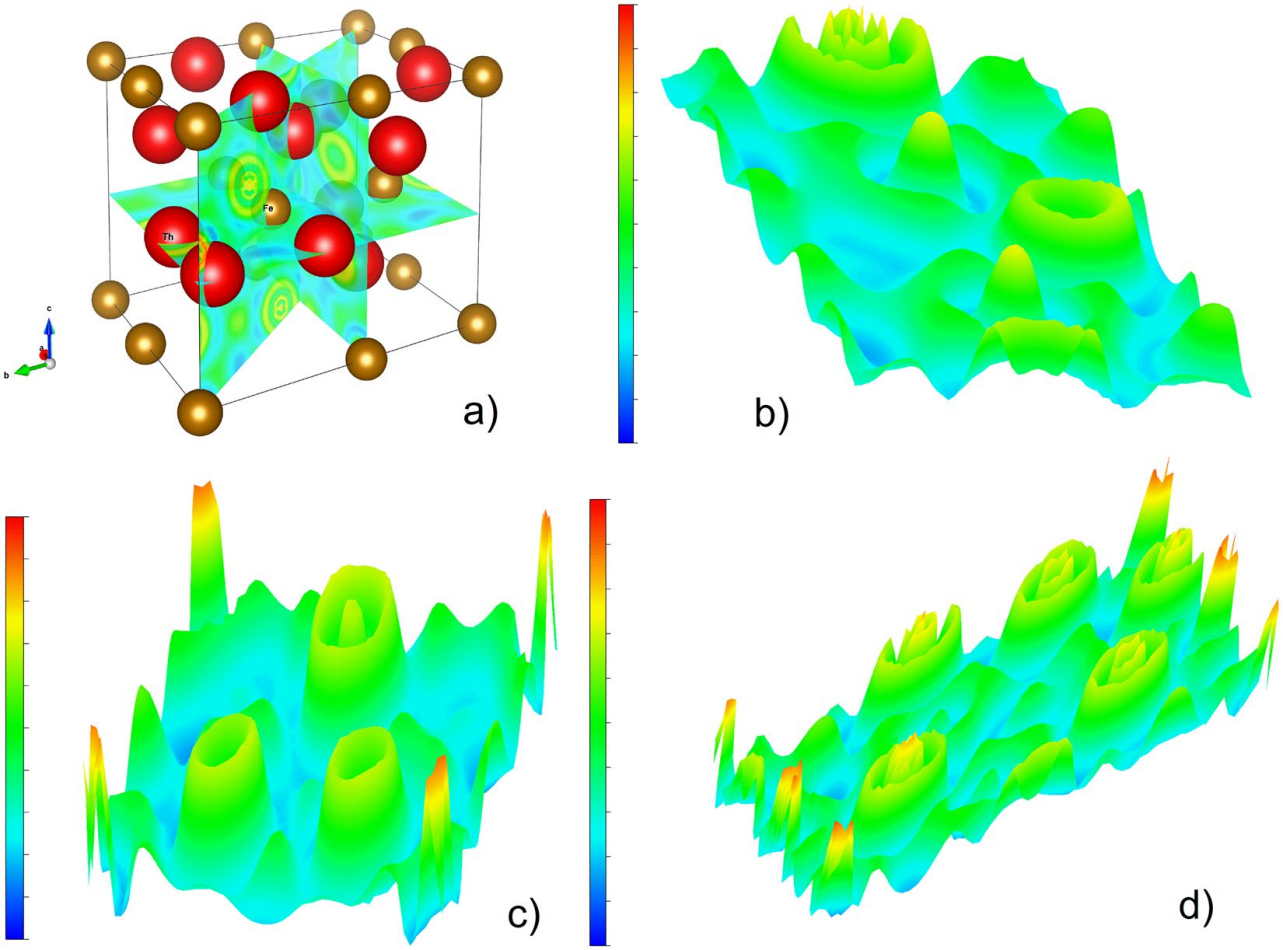
(**a**) 3D vizualizations of crystal unit cell and the electron localization function iso-surfaces cutting, in the (**b**) (001)-plane, (**c**) (010)-plane and (**d**) (110)-plane, respectively. The ELF values are bound between 0 (blue color) and 1 (red color).

Since the information about Electron Localization Function (ELF) topology is important for understanding the bonding nature of materials^40^, we have calculated ELF. There are shown the crystal unit cell together with the ELF isosurfaces cutting through the Th and Fe atoms in Fig. 8(a). The 3D vizualizations of ELF in (001)-, (010)- and (110)-plane are depicted in Fig. 8(b–d), respectively. We would like to pay attention to topological differences between regions at Th and at Fe atoms. The ELF of the Fe atoms is characterized by peaked maxima and almost spherically symmetric. High values at these maxima of about 0.78 in Fig. 8(c) and 0.82 in Fig. 8(d) evidence that electrons around the Fe cores are strongly paired and they are attractors^41^. On the other hand, the ELF of the Th atoms exhibits broader peak with a relatively low value of about 0.7, but this value still indicates a covalent bonding. The observed difference in the ELF values of the Th and Fe core regions certainly manifests the different strength of covalent bonds. Surprisingly, the ELF maximum of the Th cores is found inside external wall. As follows, the ELF around the Th atoms has anisotropic, extended volcano-like shape. It is noticed that the ELF values of the external walls are approximately 0.5–0.6, suggesting the region of delocalized electrons. Thus, the distinction of ELF values in Th_7_Fe_3_ apprises a change in the bonding properties, from strongly to weaker covalent, and to metallic character.

## Conclusions

In summary, we measured specific heat and electrical resistivity as well as performed electronic band structure calculations using FP-LAPW method for hexagonal, noncentrosymmetric Th_7_Fe_3_ compound. The measurements reveal that the studied material is a weakly electron correlated superconductor with superconducting phase transition at 1.98 ± 0.05 K. In particular, anomalous behaviour observed in *C*_*el*_(*T*)/*T*, *γ*(*H*) and *H*_*c*2_(*T*_*c*_) provides evidence for the existence of two superconducting energy gaps. Based on experimental data we also determined some fundamental thermodynamic parameters, which are gathered in Table 1.

**Table 1:** Thermodynamic parameters of Th_7_Fe_3_ in the superconducting and normal states.

Parameters (units)	Th_7_Fe_3_
*T*_*c*_ (K)	1.98 ± 0.05
${H}_{{c}_{2}}\mathrm{(0)}$ (kOe)	5.4–13 ± 0.5
*H*_*c*_(0) (Oe)	194.6
*λ*_*GL*_ (nm)	361.2–751.4
*ξ*_*GL*_(0) (nm)	13.2–15.9
*κ*_GL_	27.2–47.1
*α*	0.21
*λ*_el−ph_	0.59
Δ*C*_*p*_/*γT*_*c*_	1.21
2Δ_1_/*k*_*B*_ *T*_*c*_	3.22
2Δ_2_/*k*_*B*_ *T*_*c*_	0.76
*γ* (mJ/molK^2^)	52.7 ± 0.2
*N*(*E*_*F*_)^*exp*^ (st./eV f.u.)	22.3
*N*(*E*_*F*_)^*theo*^ (st./eV f.u.)	~20
*β* (mJ/molK^4^)	5.5
${{\rm{\Theta }}}_{D}^{LT}$ (K)	152 ± 2
${{\rm{\Theta }}}_{D}^{HT}$ (K)	215 ± 3

The electronic band structure calculation supports non-magnetic ground state of the superconductor. The theoretical partial DOS at *E*_*F*_ imply equal contributions of the 3d-electron of Fe and Th 6d-electrons to the total DOS. The mixture of these d-electrons is conjectured to be responsible for the superconductivity in Th_7_Fe_3_. There are six bands crossing the Fermi level, and the Fermi surfaces are ascribed to two bands hole-like at the *A* point but electron-like around the Γ point, two bands hole-like at the *A* point and both hole- and electron-like at the Γ point, and the two hole-like bands around both the *A* and Γ points. Two observed types of charge carriers are affected by ASOC through lowering band energies as compared with those without SOC. It is suggestive that this multiband structure may have close relation with two-gap superconductivity in the studied material. The distinct differences in both EBS and FS’s obtained without and with SOC reflect considerable effect of band splitting. Strong anisotropic properties in SBS, FS’s and ELF are ascribed to ASOC associated with noncentrosymmetric structure. With the aid of ELF data, we examined the bonding nature in Th_7_Fe_3_. It was found that there are different ELF values, corresponding to different characters of bonding. In addition to the metallic bonds, strongly covalent bonds were found around the Fe atoms but somewhat weak strength around the Th atoms. We think that the observed experimental and theoretical properties of Th_7_Fe_3_ may be beneficial in the contest of comparative investigations of noncentrosymmetric superconductors without strong electron correlation effects.

## Methods

Polycrystalline sample of Th_7_Fe_3_ was prepared from pure elements Th: 99.8% and Fe: 99.99%. A two-step synthesis was carried out using an arc-melting under a Ti-gettered purified argon atmosphere. First, the Th content was firstly melted separately and then impurities on the surface of the melted button were removed by mechanical cleaning and nitric acid etching. Next, a mixture of the stoichiometric ratio 7:3 of Th and Fe was remelted several times to insure homogeneity. The as-cast Th_7_Fe_3_ specimen was wrapped in tantalum foil, sealed into evacuated quartz tube and annealed at 800° for two weeks. The quality of the Th_7_Fe_3_ sample was checked using powder X - ray diffraction (XRD) at room temperature, utilizing an X′Pert PRO diffractometer with monochromatized CuK_α_ radiation (*λ* = 1.5406 Å) at the 2*θ* range of 10–90°. The observed Bragg peaks in the XRD pattern indicate that the studied sample is highly homogeneous, crystallized in its own type hexagonal with the space group P6_3_mc. We are able to index all observed Bragg reflections with the lattice parameters *a* = *b* = 0.9849 nm and *c* = 0.6198 nm, being comparable to those previously reported^16,42^. It is recalled that the crystal unit cell can be characterized by the three atomic positions for thorium atoms with Th_1_, Th_2_ located at two (6c) positions and Th_3_ at (2b) position and one position (6c) for iron atoms. Specific heat *C*_*p*_(*T*) and electrical resistivity *ρ*(*T*) measurements were carried out in a Quantum Design PPMS with a 3He option in the temperature range 0.4–400 K and in magnetic fields up to 2 T. The *C*_*p*_(*T*) data were collected using the relaxation-time technique and the two-tau model. Heat capacity of sample platform with very small quantity of the apiezon N cryogenic grease was measured prior to the *C*_*p*_ measurement. The given values of the specific-heat have an uncertainty of less than 5%. The *ρ*(*T*) data were measured using the standard ac four-probe method applying an alternating current of 1 mA with a frequency of 47 Hz. The gold wires used as electrical contacts were bonded with a silver paste. The error in the reported resistivity is about 10% mainly due to the presence of micro-cracks in the sample.

Theoretical results including electronic band structures, densities of states, Fermi surfaces and electron localization function were obtained from density functional theory (DFT) calculations using all-electron Full-Potential Linearized Augmented Plane Wave (FP-LAPW) method as implemented in ELK code, available under the GNU Public License^43^. The parametrization given by Perdew *et al*.^44,45^ is used for the exchange correlation potential within the Generalized Gradient Approximation (GGA). Muffin-tin radii of 2.918 a.u. and 2.334 a.u. were used for Th and Fe atoms, respectively. This corresponds to the total number of core states of 1012, total number of valence states of 566, and total number of local-orbitals of 360. We have computed total energy as a function of number of 8 × 8 × 12 Brillouin zone (BZ) mesh, while in the Fermi surfaces calculations we used 60 × 60 × 60 mesh. The self-consistent field cycles were iterated until the total energy was stable to within 1 meV. The calculations were conducted using relativistic approaches without and with spin-orbit couplings. For the latter treatment, we have included also spin polarization to look for eventual spontaneous magnetization. Electronic band structure was calculated along the high-symmetry *A* − Γ − *M* − *L* − *A* − *H* − *K* − Γ lines.
